# Potential role of a ventral nerve cord central pattern generator in forward and backward locomotion in *Caenorhabditis elegans*

**DOI:** 10.1162/netn_a_00036

**Published:** 2018-09-01

**Authors:** Erick O. Olivares, Eduardo J. Izquierdo, Randall D. Beer

**Affiliations:** Cognitive Science Program, Indiana University, Bloomington, IN, USA; Cognitive Science Program, Indiana University, Bloomington, IN, USA; School of Informatics and Computing, Bloomington, IN, USA; Cognitive Science Program, Indiana University, Bloomington, IN, USA; School of Informatics and Computing, Bloomington, IN, USA

**Keywords:** Behavioral connectomics, Network dynamics, Central pattern generator, Locomotion

## Abstract

*C. elegans* locomotes in an undulatory fashion, generating thrust by propagating dorsoventral bends along its body. Although central pattern generators (CPGs) are typically involved in animal locomotion, their presence in *C. elegans* has been questioned, mainly because there has been no evident circuit that supports intrinsic network oscillations. With a fully reconstructed connectome, the question of whether it is possible to have a CPG in the ventral nerve cord (VNC) of *C. elegans* can be answered through computational models. We modeled a repeating neural unit based on segmentation analysis of the connectome. We then used an evolutionary algorithm to determine the unknown physiological parameters of each neuron so as to match the features of the neural traces of the worm during forward and backward locomotion. We performed 1,000 evolutionary runs and consistently found configurations of the neural circuit that produced oscillations matching the main characteristic observed in experimental recordings. In addition to providing an existence proof for the possibility of a CPG in the VNC, we suggest a series of testable hypotheses about its operation. More generally, we show the feasibility and fruitfulness of a methodology to study behavior based on a connectome, in the absence of complete neurophysiological details.

## Author Summary

Despite the relative simplicity of *C. elegans*, its locomotion machinery is not yet well understood. We focus on the generation of dorsoventral body bends. Although network central pattern generators are commonly involved in animal locomotion, their presence in *C. elegans* has been questioned due to a lack of an evident neural circuit to support it. We developed a computational model grounded in the available neuroanatomy and neurophysiology, and we used an evolutionary algorithm to explore the space of possible configurations of the circuit that matched the neural traces observed during forward and backward locomotion in the worm. Our results demonstrate that it is possible for the rhythmic contraction to be produced by a circuit present in the ventral nerve cord.

## INTRODUCTION

With 302 neurons and an almost completely reconstructed connectome (Chen, Hall, & Chklovskii, 2006; Jarrell et al., 2012; Varshney, Chen, Paniagua, Hall, & Chklovskii, 2011; White et al., 1986), *Caenorhabditis elegans* is an ideal organism to study the relationship between network structure, network dynamics, and behavior. Since nearly the entire behavioral repertoire of *C. elegans* is ultimately expressed through movement, understanding the neuromechanical basis of locomotion and its modulation is especially critical as a foundation on which analyses of all other behaviors must build. Similar to other nematodes, *C. elegans* locomotes in an undulatory fashion, generating thrust by propagating dorsoventral bends along its body with a wavelength of undulation that depends on the properties of the medium through which it moves (Berri, Boyle, Tassieri, Hope, & Cohen, 2009; Fang-Yen et al., 2010; Gray & Lissmann, 1964; Lebois et al., 2012). Movement is generated by a total of 95 rhomboid body wall muscles arranged in staggered pairs along four dorsal/ventral and left/right longitudinal bundles (DR, DL, VR, and VL containing 24, 24, 24 and 23 muscles, respectively) (Sulston & Horvitz, 1977; Waterston, 1988). The neck and body are driven by 75 ventral cord motorneurons divided into eight distinct classes (dorsal 11-AS, 9-DA, 7-DB, and 6-DD and ventral 12-VA, 11-VB, 6-VC, and 13-VD). The ventral cord is interconnected by a network of chemical and electrical synapses, which are in turn activated by a set of five lateral pairs of command interneurons (AVA, AVB, AVD, AVE, and PVC) that interact with a variety of interneuronal and sensory neurons in the head (White, Southgate, Thomson, & Brenner, 1976; White et al., 1986). The motor pattern can be divided into two components that need to be understood: the generation of rhythmic alternating bends and the propagation of these bends along the body. Three hypotheses have been proposed for the generation of the rhythmic pattern (Cohen & Sanders, 2014; Gjorgjieva, Biron, & Haspel, 2014; Zhen & Samuel, 2015): (a) oscillations through stretch-receptor feedback; (b) oscillations originating from a single central pattern generator (CPG) in the head circuit; and (c) oscillations from one or more CPGs along the ventral nerve cord (VNC). To date there have been models of locomotion that range from purely neural (Bryden & Cohen, 2008; Karbowski, Schindelman, Cronin, Seah, & Sternberg, 2008; Niebur & Erdös, 1993) to purely mechanical (Majmudar, Keaveny, Zhang, & Shelley, 2012; Niebur & Erdös, 1991). They can be broadly divided into those that include a neuronal CPG in the head (Deng, Xu, Wang, Wang, & Chen, 2016; Karbowski et al., 2008; Niebur & Erdös, 1991; Sakata & Shingai, 2004; Wen et al., 2012) and those that rely primarily on sensory feedback mechanisms to generate the rhythmic pattern (Boyle, Berri, & Cohen, 2012; Boyle, Bryden, & Cohen, 2008; Bryden & Cohen, 2008; Kunert, Proctor, Brunton, & Kutz, 2017). Until now, no neuroanatomically grounded model had considered the third hypothesis: the existence of CPGs in the VNC.

Although CPGs are involved in animal locomotion in many organisms, from leech to humans (Dimitrijevic, Gerasimenko, & Pinter, 1998; Guertin, 2013; Ijspeert, 2008; Marder, Bucher, Schulz, & Taylor, 2005; Selverston, 2010), their presence in *C. elegans* has been questioned on two accounts. First, there has been evidence for the role of stretch receptors in propagating the oscillatory wave posteriorly along the body (Wen et al., 2012). However, the VNC CPGs hypothesis cannot be discarded based purely on the participation of stretch receptors. Even with their involvement, intrinsic network oscillations remain a possibility in the worm. Second, there has been no clear evidence of pacemaker neurons involved in the generation of oscillations for locomotion in *C. elegans*. Similarly, regardless of the presence or absence of pacemaker neurons, the possibility of a network oscillator is still feasible. However, work in the locomotion literature has claimed that the synaptic connectivity of the VNC does not contain evident subcircuits that could be interpreted as local CPG elements capable of spontaneously generating oscillatory activity (Wen et al., 2012). With a fully reconstructed connectome, the question of whether such subcircuits exist can be explored systematically through computational models.

**Figure 1.**
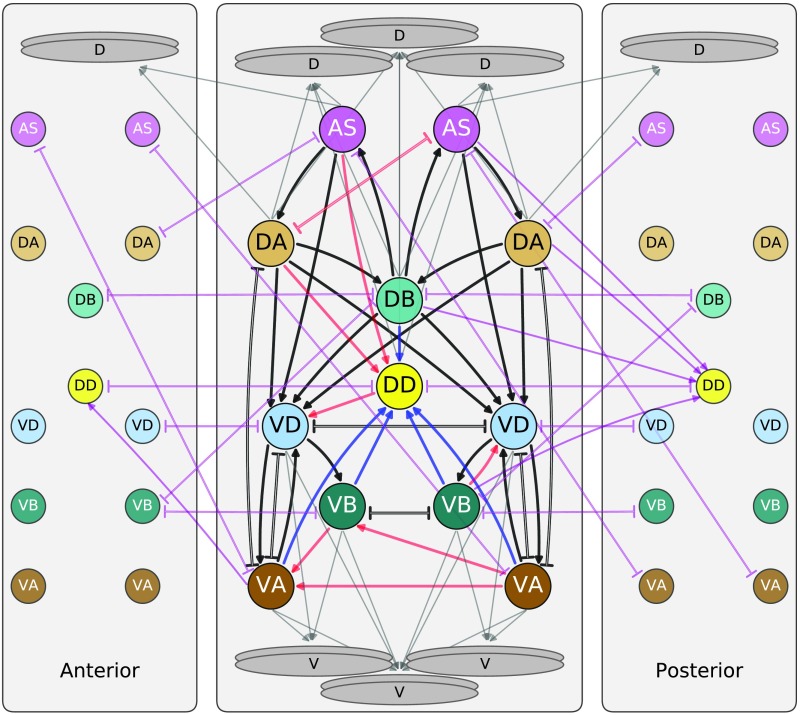
Repeating neural unit in the VNC, adapted from Haspel & O’Donovan (2011). Arrows represent chemical synapses (→). Connections ending in lines represent electrical synapses (${}_{\left|—\right|}$). Black and blue connections are anterior-posterior symmetric; red connections are not. Purple connections correspond to intersegmental synapses. Gray connections represents neuromuscular junctions.

To explore the feasibility of VNC CPGs for locomotion, we developed a computational model of a neural circuit representing one of several repeating neural units present in the VNC. Although the nematode is not segmented, a statistical analysis by Haspel & O’Donovan of the motorneurons in relation to the position of the muscles they innervate revealed a repeating neural unit along the VNC (Haspel & O’Donovan, 2011). Their statistical analysis resulted in a repeating neural unit comprising 2 DA, 1 DB, 2 AS, 2 VD, 2VA, and 1 DD motorneurons, connected by a set of chemical synapses (→) and gap junctions (${}_{\left|—\right|}$) (Figure 1). Although not all the connections in this statistically repeating neural unit are present in every one of the units of the VNC, we used it as a starting point for our study of intrinsic network oscillations. Following previous work (Izquierdo & Beer, 2013; Izquierdo & Lockery, 2010), motorneurons were modeled as isopotential nodes with the ability to produce a regenerative response, parametrized to match the full range of electrophysiological activity observed in *C. elegans* neurons (Mellem, Brockie, Madsen, & Maricq, 2008).

**Figure 2.**
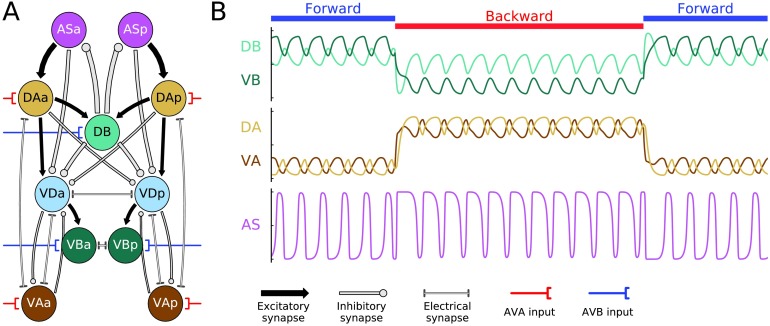
Best evolved circuit. (A) Full circuit architecture. Neurons and connections taken from VNC neural unit (see Figure 1). The type of connection is depicted with different symbols (see legend). Unknown parameters of the circuit such as the strength of the chemical synapses and sign (whether they are excitatory or inhibitory), the strength of the gap junctions, and the intrinsic parameters of the neurons were evolved. The width of the connection is proportional to the evolved strength. External input from command interneurons AVB to B-class motoneurons and AVA to A-class motoneurons are represented in blue and red, respectively. (B) Simulated neural traces of the evolved network for forward and backward locomotion. During forward locomotion, AVB input is on. During backward locomotion, AVA input is on.

The aim of this work is to explore the space of possible configurations of the repeating neural unit in the VNC that are capable of intrinsic network oscillations resembling those observed during forward and backward locomotion in the worm. However, the physiological parameters of this neural unit are largely unknown, including which chemical synapses are excitatory or inhibitory and their strengths, the strengths of the gap junctions, and the intrinsic physiological properties of the motorneurons. Therefore, we used an evolutionary algorithm to explore the configurations of the parameters that allow for an intrinsic oscillation in the neural unit so as to match the main features that have been experimentally observed in neural traces of forward and backward locomotion. As the model does not include muscles or mechanical parts of the body, we focused on the patterns in the neural traces of the A- and B-class motorneurons as a proxy for evaluating forward and backward movement. Based on observations by Kawano et al. (2011), we modeled command interneuron input to motoneurons as constant external inputs that could be switched ON or OFF (red and blue connections, Figure 2A). Finally, by running the evolutionary algorithm many times with different random seeds, we consider not just one possible configuration for the parameters of the neural unit, but as many variations as possible. As far as we are aware, this is the first attempt to explore the feasibility of CPGs in the VNC of *C. elegans*.

## MODEL AND METHODS

### Neuroanatomical Unit

We developed a model of a repeating neural unit in the VNC proposed in Haspel & O’Donovan (2011) (Figure 1). The neural unit is based on an analysis of repeating patterns of connectivity along the VNC and a re-alignment of the motorneurons along the anteroposterior axis according to positions of the muscles they innervate. The repeating neural unit proposed in their analysis includes motorneurons: AS, DA, VA, DB, VB, DD, and VD. We introduce a number of simplifying assumptions as a starting point for our investigation of the feasibility of VNC CPGs.

First, our model assumes that cells of the same class are electrophysiologically similar. All neuron classes, except for DD and DB, comprise a pair of neurons: one anterior and one posterior. The neural unit is thus anterior-posterior symmetric in terms of cells by class. In this paper, we refer to anterior and posterior cells within a unit with the suffix ‘a’ or ‘p’ appended to the neuron’s name (e.g., DAa and DAp for anterior and posterior DA cells within the neural unit).

Second, our model of the circuit introduces an anterior-posterior symmetry constraint on the connections (Figure 2A). The majority (79.4%) of connections within the neural unit are anterior-posterior symmetric (black and brown connections, Figure 1). We assumed that the parameters of the connection (whether a connection was excitatory or inhibitory and its strength for a chemical synapse, and the strength for a gap junction) were anterior-posterior class symmetric. That is, for example, the connection between AS and DA was the same for the anterior part of the circuit as it was for the posterior half of the circuit. To further reduce the number of parameters in the model for our initial search, we also omitted the 8 connections that are not anterior-posterior symmetric (red connections, Figure 1). This simplification allows us to easily constrain the activity of cells from the same class within the unit to exhibit a similar pattern of activity as has been seen experimentally (Haspel, O’Donovan, & Hart, 2010).

Third, our model omits the DD motorneuron from the circuit. In the Haspel and O’Donovan unit (Figure 1), the DD motorneuron has only one outgoing connection (i.e., DD → VD). This connection is not anterior-posterior symmetric (i.e., it connects to the anterior VD cell, but not to the posterior one). With the introduction of the anterior-posterior symmetry constraint on the connections, the DD neuron is left with no outgoing chemical synapse. Based again on preliminary experiments with the full asymmetrical circuit, we noticed that DD never showed a role in the generation of the oscillatory pattern. So as the starting point into our search for a potential VNC CPG, we omitted the DD motorneuron and its incoming connections (brown connections, Figure 1). Although DD can play an important role in driving locomotion in the physical body, because of its neuromuscular junctions, due to its lack of outgoing connections within the neural unit, it is not likely to play a role in the generation of intrinsic network oscillations.

### Neural Model

Following electrophysiological studies in *C. elegans* (Lockery & Goodman, 2009; Mellem et al., 2008) and previous modeling efforts (Izquierdo & Beer, 2013; Izquierdo & Lockery, 2010), motorneurons were modeled as isopotential nodes with the ability to produce regenerative responses, according to the following:

$$\tau_{i}\frac{dy_{i}}{dt}=-y_{i}+\overset{N}{\underset{j=1}{\sum }}w_{ji}\sigma (y_{j}+\theta_{j})+\overset{N}{\underset{k=1}{\sum }}g_{ki}(y_{k}-y_{i})+I_{i}$$ (1)

where *y*_*i*_ represent the membrane potential of the *i*th neuron relative to its resting potential, *τ*_*i*_ is the time constant, *w*_*ji*_ corresponds to the synaptic weight from neuron *j* to neuron *i*, and *g*_*ki*_ as a conductance between cell *i* and *j* (*g*_*ki*_ > 0). The model assumed chemical synapses release neurotransmitter tonically and that steady-state postsynaptic voltage is a sigmoidal function of presynaptic voltage (Kuramochi & Doi, 2017; Lindsay, Thiele, & Lockery, 2011; Wicks, Roehrig, & Rankin, 1996), *σ*(*x*) = 1/(1 + *e*^−*x*^), where *σ*(*x*) is the synaptic potential or output of the neuron. The chemical synapse has two parameters: *θ*_*j*_ is a bias term that shifts the range of sensitivity of the output function, and *w*_*ji*_ represents the strength of the chemical synapse. When the bias term is a low value, the neuron is *intrinsically inactive*: in the absence of excitatory stimuli the neuron output is off. When the bias term is a high value, the neuron is *intrinsically active*: without external inhibition the neuron’s synaptic release has a steady basal level. Unfortunately, there is no concrete evidence about the nature of electrical synapses in *C. elegans*. Until more evidence is available, and in line with previous models (Izquierdo & Beer, 2013; Kunert et al., 2017; Wicks et al., 1996), the model assumes electrical synapses are nonrectifying. The circuit was simulated using the Euler method with a time step of 0.0025.

**Figure 3.**
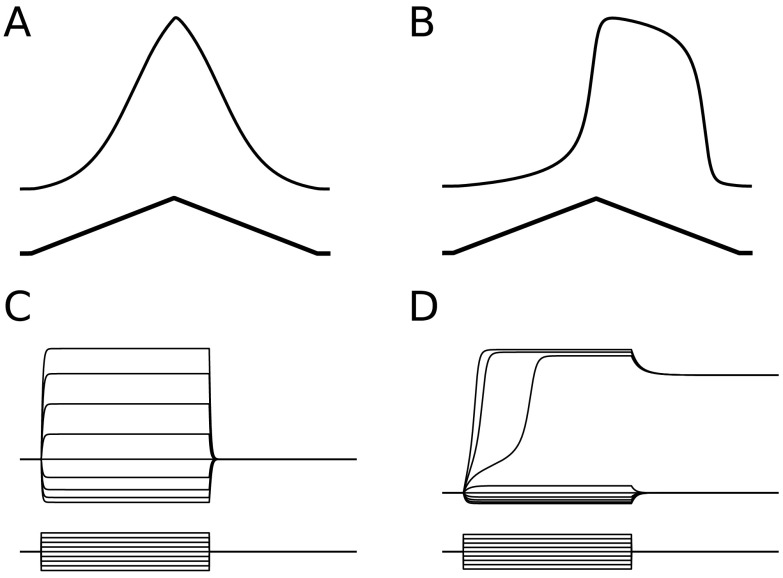
Activity of model neuron resembles electrophysiological properties of *C. elegans* neurons. We simulated the neural model in two different configurations to reproduce electrophysio logical recordings in Mellem et al. (2008): neuron AVA (left column) and neuron RMD (right column), as examples of passive and active dynamics, respectively. Parameters for the passive AVA-like neuron were *θ* = −1.8; *τ* = 0.03; *w* = 0.6. Parameters for the active RMD-like neuron were *θ* = −3.4; *τ* = 0.05; *w* = 5.1. For each of the panels, the bottom trace represents the external input and the top trace represents the output of the neuron. Responses to a depolarizing current ramp from the passive neuron configuration (A) and the active response configuration (B) of the model. Responses to a current steps for the passive neuron configuration (C) and the active response configuration (D). The active configuration reproduces the long-lived plateau potentials in response to depolarizing current steps observed in *C. elegans* neurons (Mellem et al., 2008).

Our neural model has the capacity to reproduce qualitatively the range of electrophysiological properties observed so far in *C. elegans* neurons (Lockery & Goodman, 2009; Mellem et al., 2008) (Figure 3). The model can reproduce the passive activity that has been observed in some neurons (e.g., AVA): linear voltage response to depolarizing current ramps and a graded return to resting potential in response to current steps (Figure 3A). Through the increase of the strength of the self-connection (>4, see Beer, 1995), the model is also capable of reproducing the bistable potentials found in some neurons (e.g., RMD). The voltage response to depolarizing current ramps is initially linear, but then becomes regenerative, leading to a plateau potential (Figure 3B). We also found that depolarizing current steps were sufficient to generate long-lived plateau potentials, as in RMD neurons. On cessation of the current step, the voltage relaxed to a different steady-state value from the initial resting potential (Figure 3D), also as in RMD neurons.

### Interneuron Inputs

Interneuron control of locomotion direction depends on a relatively complex interaction between electrical and chemical synapses (Kawano et al., 2011). Whereas deletion of no single command neuron disrupts forward or backward locomotion entirely, deletion of AVA and AVB produce the most significant differences in behavior (Chalfie et al., 1985). During forward locomotion, AVB activity is active, while AVA is suppressed. During backward locomotion, the opposite is true: AVB is suppressed, while AVA is active. Although modeling the command interneuron circuit with all its synapses is outside the scope of this paper, the main role of the AVB class is to influence the B-class motorneuron to turn on and off forward locomotion, whereas the main role of AVA is to influence the A-class motorneuron to turn on and off backward locomotion (Kawano et al., 2011; Qi, Garren, Shu, Tsien, & Jin, 2012; Rakowski, Srinivasan, Sternberg, & Karbowski, 2013; Zheng, Brockie, Mellem, Madsen, & Maricq, 1999). As we are not modeling the body, and therefore speed, we do not take into account results about the influence of the slope of AVA or the basal level effect on worm speed (Kato et al., 2015). Accordingly, we modeled interneuron control of locomotion as a binary external input over A- and B-class motorneurons. During the simulation of the circuit, we refer to forward locomotion when the B-class motorneuron receives external input from AVB and the A-class motorneuron does not receive input from AVA. We refer to backward locomotion when the B-class motorneuron receives no external input from AVB, and the A-class receives external input from AVA.

### Evolutionary Optimization

The parameters of the model were evolved using a genetic algorithm. The optimization algorithm was run for populations of 1000 individuals. Each individual encoded the 34 unknown parameters of the model. These unknown parameters included those that determined the physiology of each of the six neuron classes in the model: self-weight, bias and time constant, all together 18 parameters. It also included the weights and polarities of the chemical synapses and gap junctions. The neural unit has a total of 20 chemical synapses and six gap junctions. We imposed an anterior-posterior symmetry in the sign and strength of the chemical and electrical connections, reducing the total number of unknown parameters to 10 for chemical and 4 for electrical synapses. Finally, the input from the command interneurons were modeled with two parameters: the strength from AVB to B-class motorneurons and AVA to A-class motoneurons. The range for neuron time constant was set to [0.05, 2]. The range for biases, self-weights, chemical synapses, and interneuron input was set to [−20, 20]. The range for electrical synapses was set to [0, 2.5]. We ran 1,000 evolutionary algorithms with different random seeds. Each evolutionary experiment was run for 1,000 generations.

### Fitness Function

Fitness was evaluated in a simulated forward and backward assay. At the start of each assay, a circuit was presented with the forward condition (AVB input), the transients were allowed to pass for 6 units of time, and then the neural traces were evaluated for the following 20 units of time. Then the circuit’s state was reset and presented with the backward condition (AVA input). The procedure was repeated similarly, transients were allowed to pass and then neural traces were evaluated. For each locomotion direction, neural traces were evaluated using three components that capture the main three features of the *in vivo* calcium recordings for these motorneurons during forward and backward locomotion (Faumont et al., 2011; Kawano et al., 2011). Total fitness for an individual corresponded to the multiplication of the three components: *F* = *F*_1_ * *F*_2_ * *F*_3_.

### Oscillation Criterion

Intrinsic network oscillations were measured by evaluating the total change in the derivative of the output in the dominant class of neurons:

$$F_{1}=\underset{Y}{\prod }S_{Y}$$ (2)

where

$$S_{Y}=\frac{2}{A*T}\int_{0}^{T}\left|\frac{dO_{Y}}{dt}\right|dt$$ (3)

where *A* corresponds to an optimal oscillation amplitude covering roughly one third of the full output range (*A* = 0.3). Oscillations in this limited range match what has been observed experimentally (Kawano et al., 2011), which ultimately allows for one class of motorneurons to show dominance over the other one by oscillating in the top third or the bottom third of the full output range. *T* represents the simulation time length (*T* = 20 units of time). *dO*_*Y*_ represents the rate of change of the output of neurons. *S*_*Y*_ is capped at 1. The set of dominant neurons, Y, is {DB, VBa, VBp} for forward locomotion and {DAa, DAp, VAa, VAp} for backward locomotion.

### Phase Criterion

Dorsoventral out-of-phase oscillations were evaluated by measuring the difference in sign between the derivatives of the dorsal and ventral outputs, according to the following:

$$F_{2}=\underset{\left(V,D\right)}{\prod }\left(1-\frac{1}{2T}\int_{0}^{T}\left|\right.sgn(dO_{V})+sgn(dO_{D})\left|\right.\;dt\right)$$ (4)

where *dO*_*V*_ and *dO*_*D*_ represent the rate of change of the output of ventral and dorsal motorneurons, from the set {(VBa, DB), (VBp, DB)} for forward locomotion and {(VAa, DAa), (VAp, DAp)} for backward locomotion.

### Dominance Criterion

The dominance of A- and B-classes during forward and backward locomotion was assessed using three components: the minimum output value in the dominant neuron class, the maximum output value in nondominant neuron class, and the amplitude of the oscillation in the dominant neuron class:

$$F_{3}=\underset{Y}{\prod }f(m_{Y},(1-A))\;*\;\underset{X}{\prod }f(M_{X},A)\;*\;\underset{Y}{\prod }f(A_{Y},A)$$ (5)

where *m*_*Y*_ represents the minimum value in the trace of the output from neurons in the dominant class ($m_{Y}=\underset{t}{\min}O_{Y}(t)$), *M*_*X*_ represents the maximum value in the trace of the output from neurons in the nondominant class ($M_{X}=\underset{t}{\max}O_{X}(t)$), and *A*_*Y*_ represents the amplitude of the oscillation in the trace of the output from neurons in the dominant class, as given by the difference between the minimum value and the maximum value ($A_{Y}=\underset{t}{\max}O_{Y}(t)-\underset{t}{\min}O_{Y}(t)$). During forward locomotion, *Y* = {DB, VBa, VBp} and *X* = {DAa, DAp, VAa, VAp}. The opposite is true for backward locomotion. *A* corresponds to the optimal oscillation amplitude (see previous subsection). We use a normalization function *f*, which is nonzero and smooth in the range [0, 1], maximal when *x* = *x*_0_:

$$f(x,x_{0})=0.1+0.9\;\;\;\left(\frac{x}{x_{0}}\right)\;\;e^{\left(1-x/x_{0}\right)}$$ (6)

### Postsearch Filtering

When we analyzed the ensemble of best individuals, we noticed that some of the solutions showed damped oscillations. To remove such solutions, we simulated the ensemble for a much longer time period (3000 units of time) and reevaluated them under the same fitness function. For the analysis we considered only circuits with stable oscillations.

### Ablations

To understand the role of some neuron in a circuit’s operation, it is common to eliminate the neuron from the circuit entirely through ablations. Unfortunately, ablating a neuron conflates two somewhat different effects. First, the operation of a postsynaptic neuron may depend on the temporal details of the signal it receives from a presynaptic neuron. Second, a presynaptic neuron’s tonic baseline of activity might be necessary to maintain a postsynaptic neuron’s activity in its appropriate operating range. To distinguish between these two different effects, we examine the extent to which replacing the signal in one connection with a tonic input is sufficient to maintain the normal operation of the circuit.

In our analysis of the evolved CPGs, we systematically study the participation of each connection by evaluating the circuit’s performance when the connection is substituted by a constant input. As the constant input that better substitutes the dynamic input for a given connection is unknown, we evaluated each connection by using a batch of 1,000 different constant inputs when studying chemical synapses and 2,000 values for electrical synapses. From this batch of simulations, we selected the best fitness achieved in forward and backward locomotion independently. Finding a tonic input that can substitute a connection with little or no effect to the circuit’s performance suggests that the presynaptic neuron is keeping the postsynaptic neuron within its operating range, but that there is no temporal detail in the signal being transmitted. Not finding a tonic input that allows the circuit to perform normally suggests that the presynaptic neuron’s dynamical activity is crucial for the operation of the circuit. When a neuron has no effect on any of its postsynaptic neurons, then that neuron can be said to not play a role in the circuit.

## RESULTS

### VNC Neural Unit Can Intrinsically Generate Locomotion-like Oscillations

The *C. elegans* locomotion pattern can be characterized by three features that any putative CPG must exhibit. (1) *Oscillation criterion*: the A- and B-class motorneurons oscillate (Faumont et al., 2011; Haspel et al., 2010; Kawano et al., 2011); (2) *phase criterion*: the dorsal and ventral oscillations in each class of cells occur in antiphase in order to drive the alternating dorsoventral bends responsible for locomotion (Faumont et al., 2011; Kawano et al., 2011); (3) *dominance criterion*: B-class activity exceeds A-class activity during forward locomotion, whereas A-class activity exceeds B-class activity during backward locomotion (Haspel et al., 2010; Kawano et al., 2011). Each of these features become a term in the measure of locomotion performance that we optimized. In total, 1,000 optimizations with different initial random seeds were run. The best-performing circuit obtained from each run forms a solution ensemble whose properties we study in this paper.

**Figure 4.**
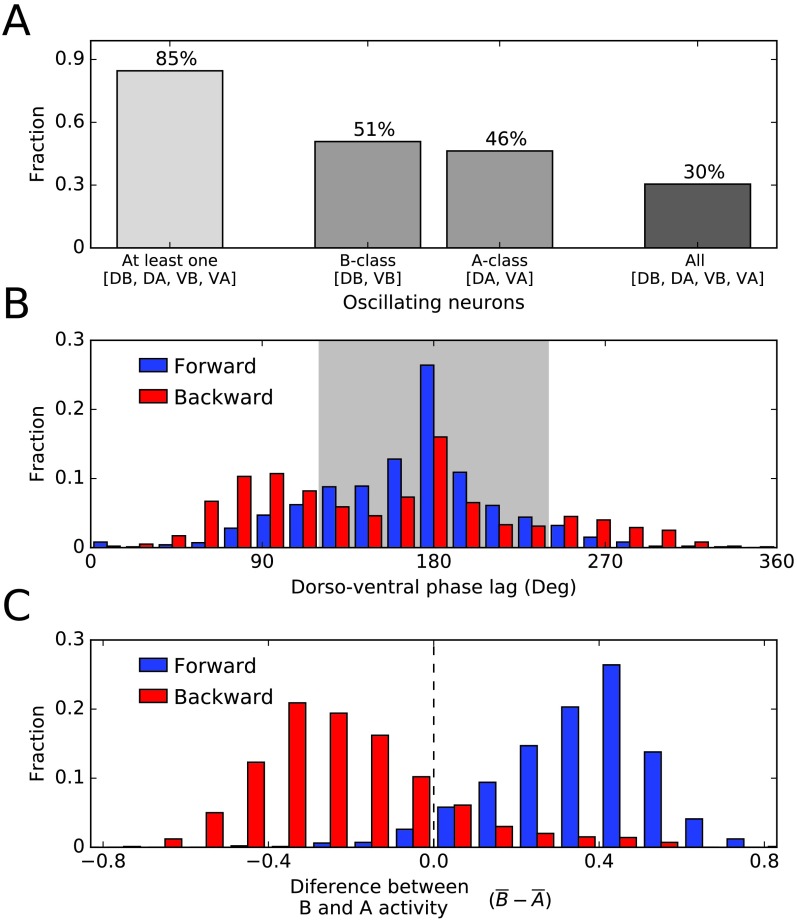
Performance of the ensemble on the individual locomotion criteria. (A) Oscillation criterion. Fraction of solutions that accomplish oscillations in one or more neurons in the network. B-class neurons were evaluated during forward locomotion and A-class during backward. Thirty percent of all solutions showed oscillations in all relevant motorneurons. (B) Phase criterion. Dorsoventral phase lag between B-class neurons for forward locomotion and A-class neurons during backward locomotion. The majority of solutions oscillate in dorsoventral antiphase. (C) Dominance criterion. Distribution of the differences in mean activity between B- and A-class neurons during forward and backward locomotion. In the majority of solutions, the A-class dominates during backward and the B-class dominates during forward.

The breakdown of the ensemble with respect to the three individual criteria is as follows. With respect to the oscillation criterion, over 80% of the ensemble exhibited oscillations in at least one neuron (Figure 4A). Specifically, 511 solutions fulfilled the criteria for forward locomotion, 463 solutions fulfilled the criteria for backward locomotion, and a total of 308 solutions achieved oscillations for both forward and backward locomotion. With respect to the phase criterion, over 78% of the circuits in the ensemble exhibited DB/VB antiphase during forward locomotion, whereas over 46% of the circuit exhibited antiphase in DA/VA neurons during backward locomotion (Figure 4B). Finally, with respect to the dominance criterion, 81% of the solutions exhibited the proper relative magnitude between the A- and B-class neurons, with B-class activity dominating during forward locomotion and A-class activity dominating during backward locomotion (Figure 4C).

**Figure 5.**
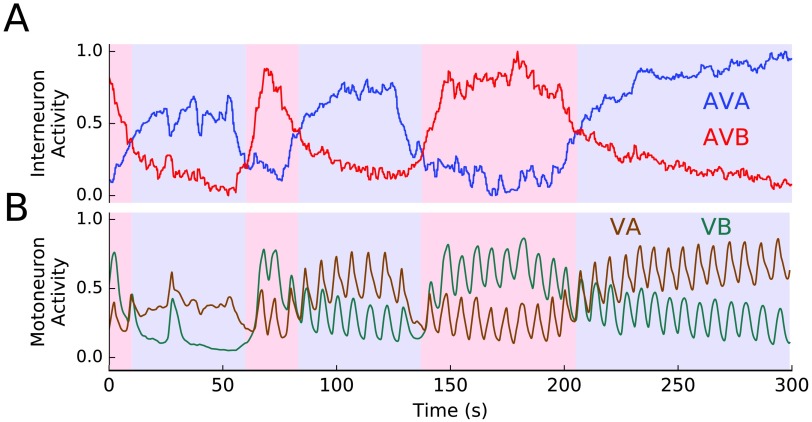
Activity of best evolved circuit during realistic stimulation by AVA and AVB command interneurons. (A) Activity of command interneurons recorded in (Kawano et al., 2011). Traces were normalized. Background color indicates forward and backward dominance depending on relative activations of AVA and AVB. (B) Traces from VA and VB neurons in the simulated circuit when stimulated by the traces in (A).

We found that over 11% of the circuits in the ensemble satisfied all three criteria, demonstrating that the repeating neural unit in the VNC is indeed capable of intrinsically generating wormlike locomotory oscillations that can be switched between a forward and backward mode by appropriate AVA and AVB command neuron input (Figure 2). Interestingly, when the model circuit is driven by realistic AVA/AVB input taken from calcium imagining of freely moving worms (see Figure 1F in Kawano et al., 2011; reproduced in Figure 5A), it produces realistic-looking motor output (compare Figure 5B to Figure 2A in Kawano et al., 2011). Specifically, the model maintains all three locomotion criteria throughout the trial, with smooth transitions in dominance between A- and B-class motorneurons that correlate with AVA and AVB activity.

### Operation of the Best Evolved Circuit

Given that the possibility of a CPG in the VNC has not been seriously considered in the literature, it is interesting to examine how this feat is achieved. Accordingly, we next analyzed the circuit with the best overall performance in the ensemble (Figure 2A). This circuit ranked 1st on the oscillation criterion, 12th on the phase criterion, and 11th on the dominance criterion. When we simulated neural traces of this circuit for forward and backward locomotion, we observed traces that matched all the main features (Faumont et al., 2011; Haspel et al., 2010; Kawano et al., 2011) (Figure 2B): (1) A- and B-class motorneurons oscillate; (2) dorsal and ventral motorneurons oscillate in antiphase; and (3) B-class baseline oscillatory activity is higher than A-class baseline oscillatory activity during forward locomotion, and the opposite is true during backward locomotion. Note that this third feature is achieved despite the activity of DB only slightly decreasing during backward locomotion. In this section, we characterize the operation of this best evolved circuit. First, we characterize a dorsal subcircuit that serves as the core oscillator driving the locomotion pattern. We then determine how these dorsal oscillations propagate to the ventral motorneurons to produce antiphase oscillations. Finally, we show how the remaining connections in the circuit fine-tune the features of the observed pattern.

#### A dorsal core subcircuit capable of oscillations.

**Figure 6.**
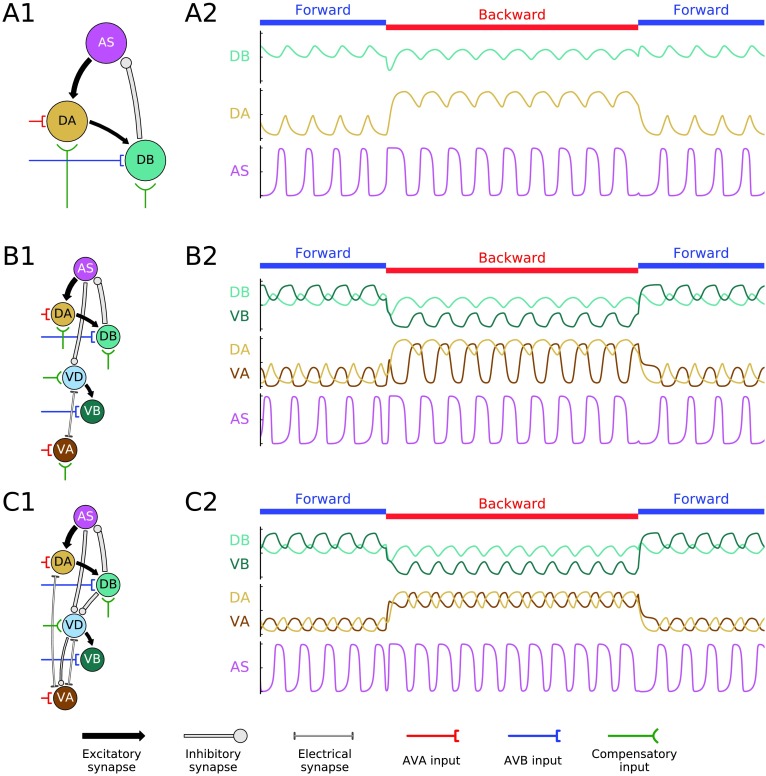
Functional analysis of the best evolved circuit. (A1) Dorsal core subcircuit. Minimal CPG comprises all three neurons that innervate dorsal muscles. Legend for the synaptic connections at the bottom of the figure. Connection width is proportional to synaptic strength. (A2) Output traces of the dorsal core subcircuit under forward and backward external inputs. Traces maintain shift in relative activation of B/A class neurons. Input from neurons AVB and AVA are shown with blue and red bars respectively. (B1) Minimal functional subcircuit. All features of the locomotion pattern are present when VD, VB, and VA are added to the dorsal core subcircuit. (B2) Simulated neural traces for the minimal functional subcircuit. Main output difference compared with the full network is the increase in amplitude in VA neuron during backward locomotion. (C1) Extended minimal circuit. (C2) Simulated neural traces for the extended minimal circuit.

How does the best circuit in the ensemble produce oscillations? Since we did not include pacemaker neurons in our model, the circuit must contain a network oscillator, that is, a subcircuit of neurons that collectively generate a rhythmic pattern through their interactions. Systematically ablating classes of neurons revealed a dorsal core subcircuit consisting of AS, DA, and DB classes capable of generating oscillations (top half of the circuit in Figure 2A). No other oscillatory subset of neuron classes was identified. In fact, further analysis demonstrated that either half of this dorsal subcircuit could oscillate in isolation, as long as appropriate tonic inputs to DA and DB were substituted for the missing neurons in order to maintain these neurons within their operating ranges (Figure 6A1). Interestingly, this dorsal core subcircuit includes the two classes of motorneurons that have been most strongly implicated in locomotion (A and B) (Chalfie et al., 1985; Haspel et al., 2010), as well as another class (AS) that has not yet been well studied experimentally.

**Figure 7.**
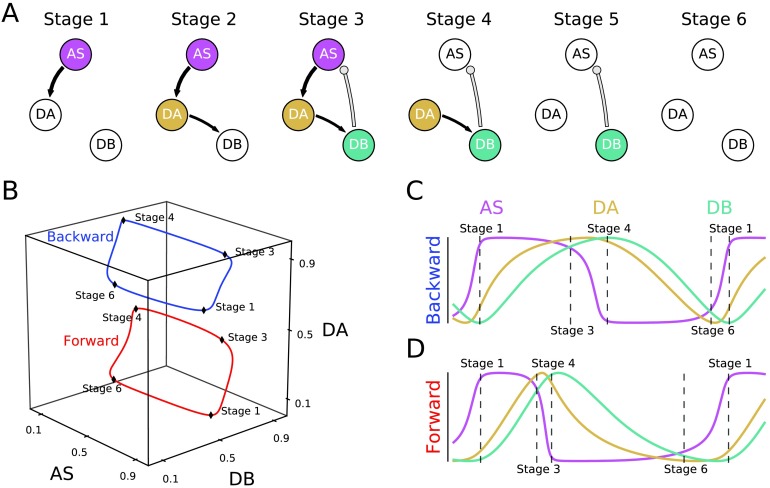
Dorsal core operation. (A) The cyclic operation of the dorsal core subcircuit can be broken down into six stages. Each stage represents a “quasi-stable” configuration of the subcircuit. Active neurons are colored; inactive neurons are shown in white. Active connections are shown; inactive connections are not shown. Dorsal core subcircuit operates similarly during forward and backward locomotion. (B) Phase plot of dorsal core neurons during backward and forward simulations. Stages 1, 3, 4, and 6 are shown with disks, according to panel (A). Dorsal core neural traces for backward (C) and forward (D). Neural traces normalized. Vertical lines mark stages 1, 3, 4, and 6 in the oscillation cycle. Although the timing of stages is the different for forward and backward, the sequence is the same.

The operation of this dorsal core subcircuit is straightforward to explain. AS is bistable (see Figure 3B) and intrinsically active. At the beginning of a locomotion cycle, AS is active and both DA and DB are inactive (Figure 7A, stage 1). Since AS excites DA, activity in the former begins to activate the latter (stage 2), which in turn excites DB, activating it as well (stage 3). But, since DB inhibits AS, its activity switches AS off (stage 4), removing the source of excitation that maintains DA and DB. As activity in DA fades (stage 5), so does activity in DB (stage 6). As AS is released from inhibition the cycle repeats, creating a CPG.

The operation of the dorsal core subcircuit is qualitatively the same for forward and backward locomotion (Figure 7B, 7C, and 7D). Although there are some small changes in the dynamics of the A- and B-class motorneurons as a result of the change of input from the command motorneurons, for the purposes of our analysis, the primary feature that changes is the level of their baseline oscillation. Both AVA and AVB evolved to excite A- and B-class motorneurons. During forward locomotion, input from AVB into B-class motorneurons produces a slight increase in the mean level of activity of B-class neurons (right shift in the location of the red limit cycle, Figure 7B), dominating the activity of the A-class neurons. The opposite is true during backward locomotion. Input from AVA into the A-class motorneurons increases the mean level of activity in the A-class activity (upward shift in the location of the blue limit cycle, Figure 7B), which ends up dominating the activity of the B-class neurons.

#### Dorsoventral coordination.

How does the dorsal oscillation spread to the ventral motorneurons in an antiphase manner? Systematic ablation of the connections from dorsal to ventral neurons demonstrates that the primary route of transmission is via the inhibitory connection from AS to VD. Like AS, VD is also bistable and intrinsically active. As AS is switched on and off by its interactions with DA and DB, it switches VD off and on, respectively, in an antiphase manner. This antiphase activity in VD is in turn propagated to VA (VD ${}_{\left|—\right|}$ VA) and to VB (VD → VB). Thus, the minimal functional subcircuit is comprised of the dorsal motorneurons AS, DA, and DB and the ventral motorneurons VD, VA, and VB along with the connections shown in Figure 6B. Note that, once again, tonic compensatory input to VD and VA is required in order for these neurons to remain within the appropriate operating range.

#### Fine-tuning the pattern.

The only significant difference remaining between the oscillation pattern of the minimal functional subcircuit (Figure 6B) and that of the full circuit (Figure 2) involves VA. As in the worm (Haspel et al., 2010; Kawano et al., 2011), during backward locomotion in the full circuit the minimum activity of VA remains quite high. However, in the minimal functional subcircuit, the minimum VA activity falls to almost zero under the same conditions (Figure 6B2). We found that we could restore the amplitude of VA activity only by including the gap junction DA ${}_{\left|—\right|}$ VA and the inhibitory chemical connection VD → VA. Interestingly, the shape and amplitude of the VA oscillation cannot be simultaneously maintained by tonic substitution alone; phasic interaction is required. With these connections (Figure 6C), the minimal functional subcircuit is capable of producing a pattern that satisfies all three locomotion criteria and is nearly identical to that of the full circuit. Finally, the contributions of the rest of the components in the full circuit were minor (see white disks in Figure 9). Substituting chemical synapses VA → VD and DA → VD for a compensatory tonic input reduced locomotion fitness to 93% and 99%, respectively. Substituting gap junctions VD ${}_{\left|—\right|}$ VD and VB ${}_{\left|—\right|}$ VB did not reduce locomotion fitness at all.

### Variations in the Ensemble

The analysis in the previous section demonstrates in detail one way in which a CPG could operate in the VNC of *C. elegans*. However, the experimental constraints are sufficiently weak at present that many other circuit configurations may also be consistent with the observed neuroanatomy, making it difficult to draw general conclusions from this single example. Fortunately, our ensemble of 110 solutions (i.e., 11% out of the 1,000 solutions obtained from optimization runs with different initial random seeds that satisfy all three locomotion criteria) represents a significant sample of the space of possibilities. In this section, we examine the operation of this entire set of solutions, with a particular focus on similarities and differences with the best evolved circuit.

#### Dorsal core subcircuit.

In the best circuit, the dorsal motorneurons AS, DA, and DB serve as the core pattern–generating subcircuit. How common is this core subcircuit in the ensemble? We found that this same set of neurons drives the locomotion pattern in 108 out of the 110 solutions (Figure 9A). As in the best circuit, AS was bistable in 83.6% of the solutions, whereas the A-class and B-class motorneurons were only bistable 3.6% of the time.

For the following analysis, we focused exclusively on the 108 solutions with a dorsal core subcircuit. Of the two solutions that did not utilize the dorsal core subcircuit to generate oscillations, one was driven by a ventral oscillator composed of the pair VA-VD (ranked 32 out of 108) and the other circuit required all neurons except for AS to produce oscillations (ranked 91 out of 108). This suggests that although the dorsal core subcircuit was an easily available solution, it is not the only possible way of producing oscillatory activity in the VNC. However, as these two solutions were atypical, and since the main purpose of our contribution is to demonstrate the possibility of a CPG in the VNC, we will not analyze them in any more detail.

**Figure 8.**
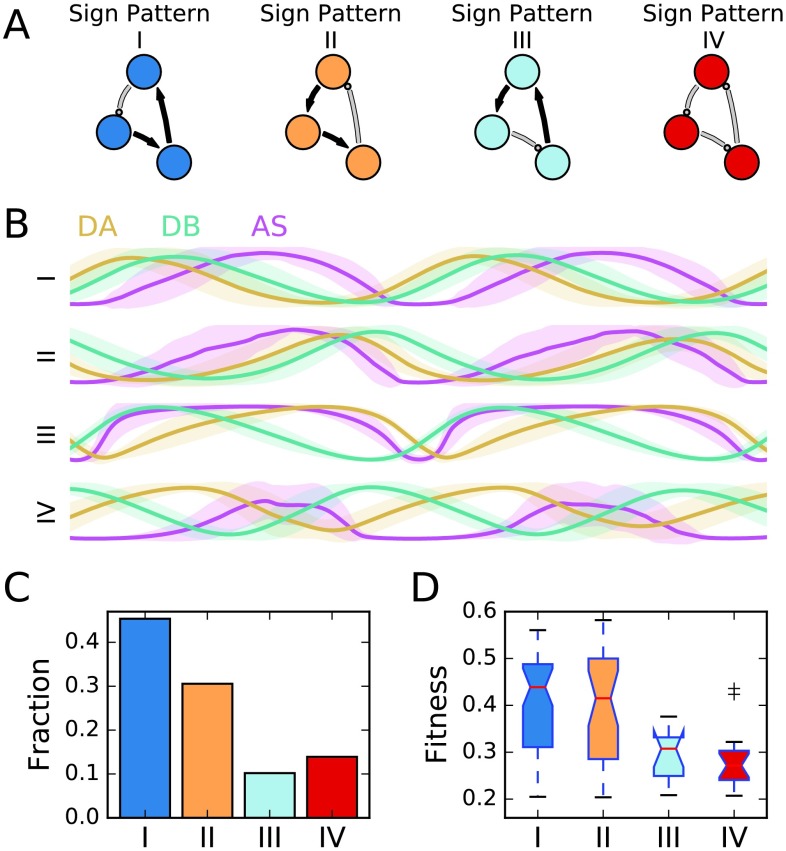
Sign patterns found in the ensemble for the dorsal core subcircuit. (A) Different patterns of signs of the connections in the dorsal core. Black arrows represent excitatory chemical synapses. Circle-ending connections represent inhibitory chemical synapses. (B) Solutions in each group show a characteristic oscillatory pattern. Traces correspond to average ±standard deviation of the normalized neuronal outputs. Two oscillation cycles are shown. (C) Proportion of the total solutions in the ensemble with each of the different dorsal core sign patterns. Sign patterns I and II represent over 75% of the solutions. (D) Fitness for the solutions according to their dorsal core sign pattern. Solutions with sign patterns I and II comprise the best performing solutions.

However, despite the near-universal appearance of dorsal core oscillators, we did find variations in the pattern of excitatory and inhibitory synapses within the ensemble. Four different sign patterns of dorsal core subcircuits were identified (Figure 8A), with the best circuit having sign pattern II. Sign patterns I, II, and III all contain two excitatory synapses and one inhibitory synapse, differing only in the placement of the latter. Sign pattern IV, in contrast, contains only inhibitory synapses. The four group of solutions exhibit neural activity patterns that are consistent within each group but differ across groups (Figure 8B). Note that DA and DB (yellow and green traces) oscillate nearly in phase in solutions with sign patterns I and II (in which the DA → DB connection is excitatory), whereas they oscillate out of phase in solutions with sign patterns III and IV (in which the same connection is inhibitory).

These four sign patterns were not equally represented across the ensemble (Figure 8C). Sign patterns I and II occur much more frequently than the other two, accounting for 75.9% of the solutions altogether. In addition to being the most represented, solutions with sign patterns I and II had on average higher fitness than solutions with sign patterns III and IV (Figure 8D). The other four logically possible combinations of excitation and inhibition among dorsal motorneurons were not observed in the ensemble.

#### Dorsoventral coordination.

**Figure 9.**
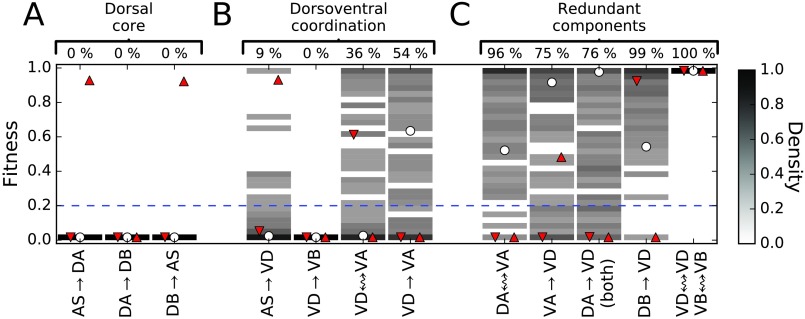
Ablation experiments. Vertical histograms show the normalized fitness distribution among the 108 solutions that evolved a dorsal core oscillator while each of the connections is exchanged for a compensatory input. The dashed line marks a minimum level of fitness of 20%. This may seem low, but the fitness is multiplicative over eight different components, such that on average each different component of fitness has above 80% performance. The percentage of solutions that maintain a fitness above this level is indicated at the top of the respective column. The white disk corresponds to the best evolved circuit. The red triangles correspond to the two solutions that did not evolve a dorsal core oscillator. The connections are divided into the following: (A) connections present in the dorsal core subcircuit; (B) connections in the minimal functional subcircuit; (C) connections responsible for minor contributions.

**Figure 10.**
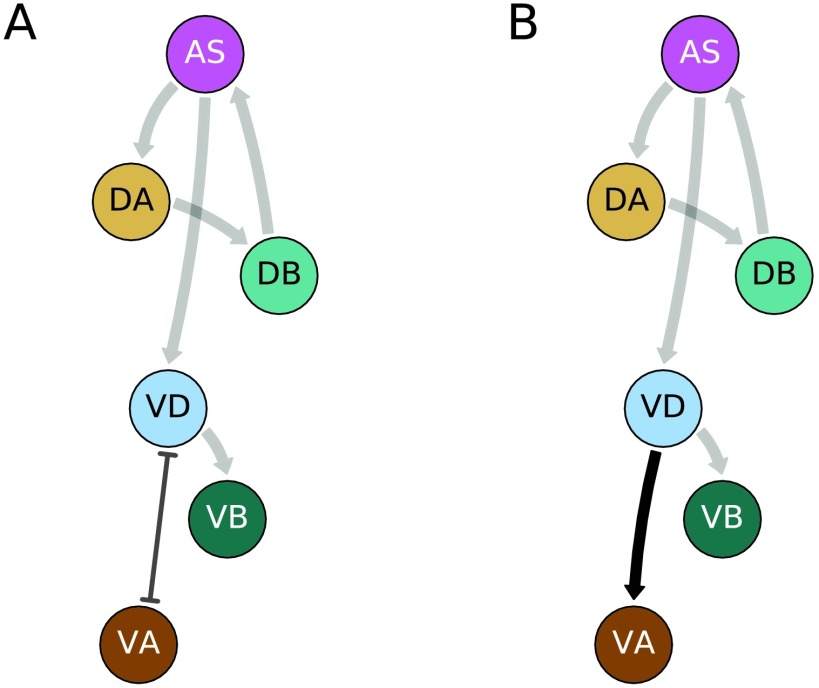
Two forms of the minimal functional circuits found in the ensemble. Common connections shown in gray. Unique connection shown in black. In both forms, the dorsal motorneurons AS, DA, and DB serve as the core pattern–generating subcircuit; dorsal oscillations propagate to ventral motorneurons in an antiphasic manner via the chemical synapse AS → VD, and from there they propagate to VB via the chemical synapse VD → VB. In one form of the solution, the information gets passed on to VA via a gap junction (A) and in others via a chemical synapse (B).

In the best circuit, dorsal oscillations propagate to ventral motorneurons in an antiphasic manner via the chemical synapse AS→VD, and then from there to VA and VB via VD ${}_{\left|—\right|}$ VA and VD → VB, respectively. How common were these patterns in the ensemble? We found that the AS → VD chemical synapse was necessary for dorsoventral coordination in 90% of the solutions and sufficient in 78% (Figure 9B). Unsurprisingly, the VD → VB chemical synapse was always essential, since no other path to VB exists in the repeating neural unit. However, two paths exist to VA: through the VD ${}_{\left|—\right|}$ VA gap junction and through the VD → VA chemical synapse. As in the best circuit, 64% of the solutions relied on the electrical connection, whereas the chemical synapse was necessary 46% of the time (some relied on both). Finally, VD was bistable in only 20% of the solutions, suggesting this feature is not essential. Therefore, the minimal functional subcircuit takes two forms in the ensemble, with the only difference being the type of connection from VD to VA (Figure 10).

#### Fine-tuning the pattern.

In the best circuit, the connections VD → VA and DA ${}_{\left|—\right|}$ VA played an important role in fine-tuning the locomotion pattern. In the ensemble, 46% of the solutions also depended on the VD → VA chemical synapse for fine-tuning. However, 96% of these solutions performed well without the DA ${}_{\left|—\right|}$ VA gap junction, suggesting that the best circuit’s use of it may have been unusual. Otherwise, the remaining connections also made only minor contributions to the solutions in the ensemble: VA → VD was unnecessary in 75% of the solutions, DA → VD was unnecessary in 76% of the solutions, DB → VD was unnecessary in 99% of the solutions, and the VD ${}_{\left|—\right|}$ VD and VB ${}_{\left|—\right|}$ VB gap junctions were always unnecessary (Figure 9C).

### Minimal Functional Subcircuit Present in Connectome

**Figure 11.**
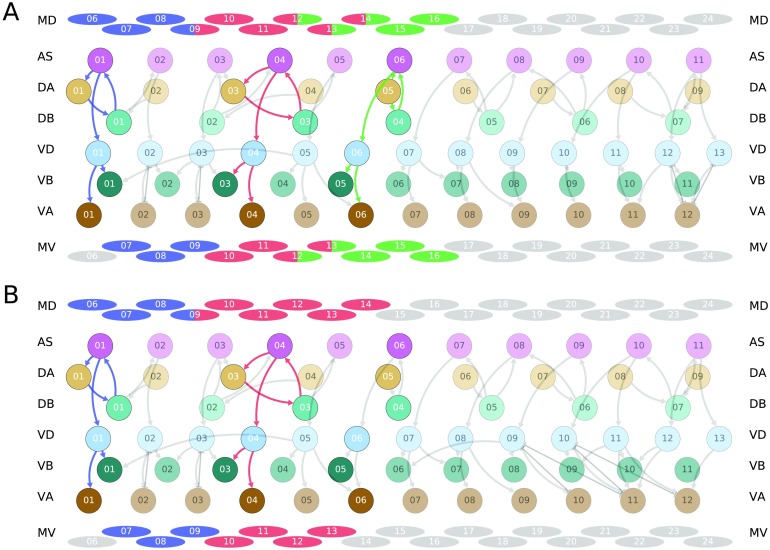
Minimal functional subcircuit present in connectome. The circuit identified in the analysis exists in three places in the anterior section of the connectome of the *C. elegans* hermaphrodite (A) and twice in the anterior section of the male connectome (B). Each functional subcircuit is shown with a different color (e.g., blue, red, and green). The muscles innervated by these subcircuits are colored accordingly. Neurons and muscles are depicted along the VNC according to their sequential order (not exact body position). Light shaded connections correspond to synapses that were identified as important for the minimal functional subcircuit along the rest of the VNC but that do not form a complete subcircuit. For clarity, all other chemical synapses and gap junctions are not shown.

It is important to recall that the repeating neural unit upon which we based our model (Figure 1) is only a statistical summary of the VNC (Haspel & O’Donovan, 2011). How well do the key components that we have identified map onto the actual neuroanatomy? To answer this question, we examined the most recent reconstructions of both the hermaphrodite and male nematodes (Jarrell et al., 2012; M. Xu et al., 2013) for the existence of the two forms of the minimal functional subcircuit found in the ensemble (Figure 10). We found that all of the key components of one of the forms of the minimal functional subcircuit occur together in three different places in the hermaphrodite (Figure 11A) and twice in the male connectome (Figure 11B). This includes the AS-DA-DB dorsal core oscillator, the AS → VD connection responsible for antiphase dorsoventral propagation, and the VD → VA and VD → VB connections that propagate the oscillation to the remaining ventral motorneurons. From this result, we made three main observations. First, although the most anterior instance aligns perfectly with the perimotor segmentation proposed by (Haspel & O’Donovan, 2011), the other two do not: they include neurons across adjacent units of the VNC. This suggests CPGs might be occurring across the repeating neural units originally proposed. Second, the motorneurons in each of the three instances of the minimal functional circuit innervate muscles that are relatively close to each other. Such an alignment could provide a solid foundation for driving movement within a significant segment of the body. Finally, altogether the three subcircuits innervate muscles spanning the full anterior body (Figure 11). This makes sense given the important role that stretch receptors have been shown to play in propagating the wave posteriorly during forward locomotion (Wen et al., 2012).

## DISCUSSION

The goal of this work was to test whether the VNC is capable of intrinsically generating oscillations that match what has been observed in A- and B-class motorneurons during forward and backward locomotion (Faumont et al., 2011; Haspel et al., 2010; Kawano et al., 2011). To address this, we applied evolutionary algorithms to systematically search for circuits that could generate intrinsic network oscillations within the worm’s VNC. We demonstrate for the first time that CPGs that match some of the most salient features of forward and backward neural traces are possible in the VNC, given what we know about the neuroanatomy and neurophysiology of *C. elegans*. Although we have made a number of simplifying assumptions in order to develop a neuroanatomically grounded computational model of the ventral nerve cord, these do not undermine our main result: that a VNC CPG for locomotion is feasible. Our modeling approach was deliberately minimal given our goal. Adding omitted chemical synapses, gap junctions, and motorneurons, removing the anterior-posterior symmetry constraint on the connections in the neural unit, and including additional electrophysiological details from the motorneurons and command interneurons has the potential to enhance the richness of available mechanisms for reproducing matching oscillatory activity.

Analysis of the best evolved circuit revealed a dorsal core subcircuit composed of an AS, a DA, and a DB motorneuron, altogether responsible for the generation of the oscillation. Through the use of our optimization methodology, we also revealed a number of different sign patterns in which the dorsal core oscillator could be instantiated. Although much less common, there were two other kinds of solutions found in the ensemble that were different from dorsal core subcircuits: solutions that generated oscillations through a ventral core comprising VA and VD, and a solution that required all motorneurons except AS and DD to produce oscillations. Our analysis also revealed a minimal functional circuit that included a VD, a VA, and a VB motorneuron in addition to the dorsal core, which exhibited all the main features observed in the worm’s neural traces. Interestingly, analysis of the ensemble of successful solutions revealed that the minimal functional circuit was one of the most common solutions. Based on our findings from the model, we returned to the connectome reconstructions and found multiple instances of this subcircuit in the hermaphrodite and male.

Given the existence of subcircuits in the VNC capable of producing backward-forward oscillatory activity, our model makes a set of testable predictions for the worm. Short of being able to suppress stretch-receptor activity during locomotion, to see the extent to which the worm can move forward, one of the key experiments that emerged from our analysis was to selectively suppress activity in dorsal-only or ventral-only motorneurons. Based on the role of the dorsal core subcircuit, our model suggests that suppressing only ventral motorneurons would have a smaller effect than suppressing only dorsal motorneurons. A second key experimental effort involves characterizing the physiology of the AS motorneuron. If a dorsal core is present in the worm, then our model suggests AS is likely to be a bistable motorneuron (Mellem et al., 2008), but not DA or DB. A third key experiment involves disrupting the chemical synapse from AS to VD. Regardless of how oscillations are generated, if the pattern of activity is present in the dorsal motorneurons, our model suggests it can be spread to ventral motorneurons through the connection between AS and VD to produce dorsoventral antiphase patterns of activity. Finally, based on our analysis, network oscillators are most feasible in the anterior portion of the VNC. Although further investigations would be required, our evolutionary experiments did not identify a CPG subcircuit in the posterior half of the VNC. In the theory-experiment cycle, results from additional experiments in the worm will help add additional constraints to our model.

Three hypotheses for the generation of oscillations for locomotion in *C. elegans* have been postulated (Cohen & Sanders, 2014; Gjorgjieva et al., 2014; Zhen & Samuel, 2015): (a) Via stretch-receptor feedback; (b) Via a single CPG in the head circuit; and (c) Via one or more CPGs along the VNC. Until now, only the first of these two hypotheses had been demonstrated using neuroanatomically grounded computational models (Boyle et al., 2012, 2008; Bryden & Cohen, 2008; Deng et al., 2016; Karbowski et al., 2008; Kunert et al., 2017; Niebur & Erdös, 1991, 1993; Sakata & Shingai, 2004; Wen et al., 2012). By demonstrating that intrinsic network oscillations in the ventral nerve cord are possible, we are elevating the previously neglected VNC CPG hypothesis to the level of feasibility of the other two more commonly discussed locomotion hypotheses. Ultimately, it is important to keep in mind that these and other related hypotheses are not mutually exclusive: the existence of a network oscillator in the VNC is compatible with many of the other mechanisms that have been suggested to operate during locomotion. First, a CPG in the worm’s head could entrain the CPGs in the VNC. Recent observations of different oscillatory rhythms in the head and the rest of the body suggests this is indeed a possibility (Fouad et al., 2017). Although we found one set of motifs that work as a CPG and was present in the anterior half of the worm, it is possible that there are other CPG motifs or reflexive pattern generators that exist in the rest of the body, including some that we have not found yet. Second, stretch-receptor feedback (Wen et al., 2012) could modulate intrinsic network oscillations from CPGs in the ventral nerve cord, effectively generating different oscillating frequencies when the worm is placed in different media (Berri et al., 2009; Fang-Yen et al., 2010; Lebois et al., 2012). Third, signals from upstream command interneurons and the interunit electrical coupling within the VNC could help coordinate multiple CPGs in the VNC and help modulate their intrinsic network oscillations (T. Xu et al., 2017). Finally, if pacemaker neurons exist in the ventral nerve cord, they could aid in the maintenance of intrinsic network oscillations within a neural unit (Gao et al., 2017).

Ultimately, understanding how neural circuits produce behavior requires an understanding of the complete brain-body-environment system (Chiel & Beer, 1997). Although we have modeled the worm’s body in previous contributions (Izquierdo & Beer, 2015), in the current model, only motorneurons were modeled. In the worm, these motorneurons are connected to the muscles through known neuromuscular junctions, which in turn drive the worm mechanically to produce thrust against the surface of its immediate environment. For any of the solutions revealed in our study to drive locomotion, the strengths of the neuromuscular junction would have to be tuned to effectively modulate the muscles. Furthermore, for the wave to travel posteriorly along the VNC, the subcircuits would have to coordinate with each other. This could be done via chemical synapses and gap junctions within the nervous system, or through the use of stretch receptors, via the mechanical movement of the body, or a combination of both. Whether forward and backward CPG-driven locomotion is feasible is unknown, but we are now in a position to test these ideas by integrating these circuits in a neuromechanical model of locomotion in the worm.

## AUTHOR CONTRIBUTIONS

Erick O. Olivares: Data curation; Formal analysis; Methodology; Software; Visualization; Writing – original draft; Writing – review & editing. Eduardo J. Izquierdo: Conceptualization; Formal analysis; Funding acquisition; Investigation; Methodology; Project administration; Supervision; Visualization; Writing – original draft; Writing – review & editing. Randall D. Beer: Conceptualization; Formal analysis; Funding acquisition; Investigation; Methodology; Project administration; Resources; Software; Supervision; Writing – original draft; Writing – review & editing.

## FUNDING INFORMATION

The work in this paper was supported in part by NSF grant IIS-1524647.

## TECHNICAL TERMS

Connectome: Comprehensive map of neural connections of an organism. In *C. elegans*, the connectome is known at a cellular level.
Dorsoventral bends: On agar, *C. elegans* lay on their side and generate thrust by propagating dorsoventral bends along its body.
Central pattern generator: Neural network that can produce rhythmic output without sensory feedback as a result of either network interactions or pacemaker cells.
Ventral nerve cord: Part of the nervous system that runs down the ventral plane of the organism. In *C. elegans*., a set of motorneurons.
Evolutionary algorithm: Optimization algorithm inspired by biological evolution. Candidate solutions play the role of individuals in the evolving population.
Fitness function: Mathematical expression that summarizes how close a given solution is to achieving the objective in the optimization algorithm.

## References

[bib1] Beer R. D. (1995). On the dynamics of small continuous-time recurrent neural networks. Adaptive Behavior, 3(4), 469–509.

[bib2] Berri S., Boyle J. H., Tassieri M., Hope I. A., & Cohen N. (2009). Forward locomotion of the nematode *C. elegans* is achieved through modulation of a single gait. HFSP Journal, 3(3), 186–193. 19639043 10.2976/1.3082260 PMC2714959

[bib3] Boyle J. H., Berri S., & Cohen N. (2012). Gait modulation in *C. elegans*: an integrated neuromechanical model. Frontiers in Computational Neuroscience, 6, 10 10.3389/fncom.2012.00010 22408616 PMC3296079

[bib4] Boyle J. H., Bryden J., & Cohen N. (2008). An integrated neuro-mechanical model of *C. elegans* forward locomotion. In Ishikawa M., Doya K., Miyamoto H., & Yamakawa T. (Eds.), Neural Information Processing. ICONIP 2007. Lecture Notes in Computer Science (pp. 37–47). Berlin, Heidelberg: Springer.

[bib5] Bryden J., & Cohen N. (2008). Neural control of *Caenorhabditis elegans* forward locomotion: The role of sensory feedback. Biological Cybernetics, 98(4), 339–351. 18350313 10.1007/s00422-008-0212-6

[bib6] Chalfie M., Sulston J. E., White J. G., Southgate E., Thomson J. N., & Brenner S. (1985). The neural circuit for touch sensitivity in *Caenorhabditis elegans*. Journal Neuroscience, 5(4), 956–964. 3981252 10.1523/JNEUROSCI.05-04-00956.1985 PMC6565008

[bib7] Chen B., Hall D., & Chklovskii D. (2006). Wiring optimization can relate neuronal structure and function. Proceedings National Academy of Sciences of the United States of America, 103(12), 4723–4728. 10.1073/pnas.0506806103 PMC1550972 16537428

[bib8] Chiel H. J., & Beer R. D. (1997). The brain has a body: Adaptive behavior emerges from interactions of nervous system, body and environment. Trends in Neurosciences, 20(12), 553–557. 9416664 10.1016/s0166-2236(97)01149-1

[bib9] Cohen N., & Sanders T. (2014). Nematode locomotion: dissecting the neuronal–environmental loop. Current Opinion Neurobiology, 25, 99–106. 10.1016/j.conb.2013.12.003 24709607

[bib10] Deng X., Xu J.-X., Wang J., Wang G.-Y., & Chen Q.-S. (2016). Biological modeling the undulatory locomotion of *C. elegans* using dynamic neural network approach. Neurocomputing, 186, 207–217.

[bib11] Dimitrijevic M. R., Gerasimenko Y., & Pinter M. M. (1998). Evidence for a spinal central pattern generator in humans. Annals of the New York Academy Sciences, 860(1), 360–376. 10.1111/j.1749-6632.1998.tb09062.x 9928325

[bib12] Fang-Yen C., Wyart M., Xie J., Kawai R., Kodger T., Chen S., … Samuel A. D. (2010). Biomechanical analysis of gait adaptation in the nematode *Caenorhabditis elegans*. Proceedings of the National Academy of Sciences United States America, 107(47), 20323–20328. 10.1073/pnas.1003016107 PMC2996704 21048086

[bib13] Faumont S., Rondeau G., Thiele T. R., Lawton K. J., McCormick K. E., Sottile M., … others (2011). An image-free opto-mechanical system for creating virtual environments and imaging neuronal activity in freely moving *Caenorhabditis elegans*. PLoS One, 6(9), e24666 10.1371/journal.pone.0024666 21969859 PMC3182168

[bib14] Fouad A. D., Teng S., Mark J. R., Liu A., Ji H., Cornblath E., … Fang-Yen C. (2017). Distributed rhythm generators underlie *Caenorhabditis elegans* forward locomotion. bioRxiv. 10.1101/141911 PMC5780042 29360037

[bib15] Gao S., Guan S. A., Fouad A. D., Meng J., Huang Y.-C., Li Y., … Zhen M. (2017). Excitatory motor neurons are local central pattern generators in an anatomically compressed motor circuit for reverse locomotion. bioRxiv. 10.1101/135418

[bib16] Gjorgjieva J., Biron D., & Haspel G. (2014). Neurobiology of *Caenorhabditis elegans* locomotion: Where do we stand? Bioscience, 64(6), 476–486. 26955070 10.1093/biosci/biu058 PMC4776678

[bib17] Gray J., & Lissmann H. (1964). The locomotion of nematodes. Journal of Experimental Biology, 41, 135–154. 14161604 10.1242/jeb.41.1.135

[bib18] Guertin P. A. (2013). Central pattern generator for locomotion: Anatomical, physiological, and pathophysiological considerations. Frontiers in Neurology, 3, 183 10.3389/fneur.2012.00183 23403923 PMC3567435

[bib19] Haspel G., & O’Donovan M. J. (2011). A perimotor framework reveals functional segmentation in the motoneuronal network controlling locomotion in *Caenorhabditis elegans*. Journal of Neuroscience, 31(41), 14611–14623. 21994377 10.1523/JNEUROSCI.2186-11.2011 PMC3221003

[bib20] Haspel G., O’Donovan M. J., & Hart A. C. (2010). Motoneurons dedicated to either forward or backward locomotion in the nematode *Caenorhabditis elegans*. Journal of Neuroscience, 30(33), 11151–11156. 20720122 10.1523/JNEUROSCI.2244-10.2010 PMC2945236

[bib21] Ijspeert A. J. (2008). Central pattern generators for locomotion control in animals and robots: A review. Neural Networks, 21(4), 642–653. 18555958 10.1016/j.neunet.2008.03.014

[bib22] Izquierdo E. J., & Beer R. D. (2013). Connecting a connectome to behavior: An ensemble of neuroanatomical models of *C. elegans* klinotaxis. PLoS Computational Biology, 9(2), e1002890 10.1371/journal.pcbi.1002890 23408877 PMC3567170

[bib23] Izquierdo E. J., & Beer R. D. (2015). An integrated neuromechanical model of steering in *C elegans*. InAndrews P. (Eds.), Proceedings of the European conference on artificial life 2015, ECAL 2015 (pp. 199–206). Cambridge, MA: MIT Press.

[bib24] Izquierdo E. J., & Lockery S. R. (2010). Evolution and analysis of minimal neural circuits for klinotaxis in *Caenorhabditis elegans*. Journal of Neuroscience, 30(39), 12908–12917. 20881110 10.1523/JNEUROSCI.2606-10.2010 PMC3422662

[bib25] Jarrell T. A., Wang Y., Bloniarz A. E., Brittin C. A., Xu M., Thomson J. N., … Emmons S. W. (2012). The connectome of a decision-making neural network. Science, 337(6093), 437–444. 22837521 10.1126/science.1221762

[bib26] Karbowski J., Schindelman G., Cronin C. J., Seah A., & Sternberg P. W. (2008). Systems level circuit model of *C. elegans* undulatory locomotion: Mathematical modeling and molecular genetics. Journal of Computational Neuroscience, 24(3), 253–276. 17768672 10.1007/s10827-007-0054-6

[bib27] Kato S., Kaplan H. S., Schrödel T., Skora S., Lindsay T. H., Yemini E., … Zimmer M. (2015). Global brain dynamics embed the motor command sequence of caenorhabditis elegans. Cell, 163(3), 656–669. 26478179 10.1016/j.cell.2015.09.034

[bib28] Kawano T., Po M. D., Gao S., Leung G., Ryu W. S., & Zhen M. (2011). An imbalancing act: Gap junctions reduce the backward motor circuit activity to bias *C. elegans* for forward locomotion. Neuron, 72(4), 572–586. 22099460 10.1016/j.neuron.2011.09.005

[bib29] Kunert J., Proctor J., Brunton S., & Kutz J. (2017). Spatiotemporal feedback and network structure drive and encode *Caenorhabditis elegans* locomotion. PLoS Computational Biology, 13(1), e1005303 10.1371/journal.pcbi.1005303 28076347 PMC5226684

[bib30] Kuramochi M., & Doi M. (2017). A computational model based on multi-regional calcium imaging represents the spatio-temporal dynamics in a *Caenorhabditis elegans* sensory neuron. PLoS One, 12(1), e0168415 10.1371/journal.pone.0168415 28072834 PMC5224993

[bib31] Lebois F., Sauvage P., Py C., Cardoso O., Ladoux B., & Meglio P. H. J. (2012). Locomotion control of *Caenorhabditis elegans* through confinement. Biophysical Journal, 102(12), 2791–2798. 22735529 10.1016/j.bpj.2012.04.051 PMC3379027

[bib32] Lindsay T. H., Thiele T. R., & Lockery S. R. (2011). Optogenetic analysis of synaptic transmission in the central nervous system of the nematode *Caenorhabditis elegans*. Nature Communications, 2(306). 10.1038/ncomms1304 PMC3935721 21556060

[bib33] Lockery S. R., & Goodman M. B. (2009). The quest for action potentials in *C. elegans* neurons hits a plateau. Nature Neuroscience, 12(4), 377–378. 19322241 10.1038/nn0409-377 PMC3951993

[bib34] Majmudar T., Keaveny E., Zhang J., & Shelley M. (2012). Experiments and theory of undulatory locomotion in a simple structured medium. Journal of the Royale Society Interface, 9(73), 1809–1823. 10.1098/rsif.2011.0856 PMC3385758 22319110

[bib35] Marder E., Bucher D., Schulz D. J., & Taylor A. L. (2005). Invertebrate central pattern generation moves along. Current Biology, 15(17), R685–R699. 16139202 10.1016/j.cub.2005.08.022

[bib36] Mellem J. E., Brockie P. J., Madsen D. M., & Maricq A. V. (2008). Action potentials contribute to neuronal signaling in *C. elegans*. Nature Neuroscience, 11(8), 865–867. 18587393 10.1038/nn.2131 PMC2697921

[bib37] Niebur E., & Erdös P. (1991). Theory of the locomotion of nematodes: Dynamics of undulatory progression on a surface. Biophysical Journal, 60(5), 1132–1146. 19431807 10.1016/S0006-3495(91)82149-X PMC1260169

[bib38] Niebur E., & Erdös P. (1993). Theory of the locomotion of nematodes: Control of the somatic motor neurons by interneurons. Mathematical Bioscience, 118(1), 51–82. 10.1016/0025-5564(93)90033-7 8260760

[bib39] Qi Y. B., Garren E. J., Shu X., Tsien R. Y., & Jin Y. (2012). Photo-inducible cell ablation in *Caenorhabditis elegans* using the genetically encoded singlet oxygen generating protein minisog. Proceedings of the National Academy of Sciences, 109(19), 7499–7504. 10.1073/pnas.1204096109 PMC3358873 22532663

[bib40] Rakowski F., Srinivasan J., Sternberg P., & Karbowski J. (2013). Synaptic polarity of the interneuron circuit controlling *C. elegans* locomotion. Frontiers of Computational Neuroscience, 7, 128 10.3389/fncom.2013.00128 PMC3788333 24106473

[bib41] Sakata K., & Shingai R. (2004). Neural network model to generate head swing in locomotion of *Caenorhabditis elegans*. Network, 15(3), 199–216. 15468735

[bib42] Selverston A. I. (2010). Invertebrate central pattern generator circuits. Philosophical Transactions of the Royal Society London B, 365(1551), 2329–2345. 10.1098/rstb.2009.0270 PMC2894947 20603355

[bib43] Sulston J., & Horvitz H. (1977). Post-embryonic cell lineages of the nematode, *Caenorhabditis elegans*. Developmental Biology, 56(1), 110–156. 838129 10.1016/0012-1606(77)90158-0

[bib44] Varshney L., Chen B., Paniagua E., Hall D., & Chklovskii D. (2011). Structural properties of the *Caenorhabditis elegans* neuronal network. PLoS Computational Biology, 7(2), e1001066 10.1371/journal.pcbi.1001066 21304930 PMC3033362

[bib45] Waterston R. (1988). Muscle. In Wood W. (Ed.), The nematod *C. elegans* (pp. 281–335). New York: Cold Spring Harbor Laboratory Press.

[bib46] Wen Q., Po M. D., Hulme E., Chen S., Liu X., Kwok S. W., … others (2012). Proprioceptive coupling within motor neurons drives *C. elegans* forward locomotion. Neuron, 76(4), 750–761. 23177960 10.1016/j.neuron.2012.08.039 PMC3508473

[bib47] White J., Southgate E., Thomson J., & Brenner S. (1976). The structure of the ventral nerve cord of *Caenorhabditis elegans*. Philosophical Transactions of the Royal Society London B, 275(938), 327–348. 10.1098/rstb.1976.0086 8806

[bib48] White J., Southgate E., Thomson J., & Brenner S. (1986). The structure of the nervous system of the nematode *Caenorhabditis elegans*. Philosophical Transactions of the Royal Society London B, 275(938), 327–348. 10.1098/rstb.1976.0086 8806

[bib49] Wicks S. R., Roehrig C. J., & Rankin C. H. (1996). A dynamic network simulation of the nematode tap withdrawal circuit: predictions concerning synaptic function using behavioral criteria. Journal of Neuroscience, 16(12), 4017–4031. 8656295 10.1523/JNEUROSCI.16-12-04017.1996 PMC6578605

[bib50] Xu M., Jarrell T. A., Wang Y., Cook S. J., Hall D. H., & Emmons S. W. (2013). Computer assisted assembly of connectomes from electron micrographs: Application to *Caenorhabditis elegans*. PLoS One, 8(1), e54050 10.1371/journal.pone.0054050 23342070 PMC3546938

[bib51] Xu T., Huo J., Shao S., Po M., Kawano T., Lu Y., … Wen Q. (2017). A descending pathway facilitates undulatory wave propagation in *Caenorhabditis elegans* through gap junctions. bioRxiv. 10.1101/131490 PMC5948959 29686107

[bib52] Zhen M., & Samuel A. D. (2015). *C. elegans* locomotion: Small circuits, complex functions. Current Opinion Neurobiology, 33, 117–126. 10.1016/j.conb.2015.03.009 25845627

[bib53] Zheng Y., Brockie P. J., Mellem J. E., Madsen D. M., & Maricq A. V. (1999). Neuronal control of locomotion in *C. elegans* is modified by a dominant mutation in the glr-1 ionotropic glutamate receptor. Neuron, 24(2), 347–361. 10571229 10.1016/s0896-6273(00)80849-1

